# A systematic review and meta-analysis of the effects of walking training on cardiorespiratory fitness in cancer patients

**DOI:** 10.3389/fonc.2026.1852397

**Published:** 2026-06-26

**Authors:** Weikai Wang, Sujie Mao, Zijun Yu, Tao Liu, Zihan Song, Fengyu Wu

**Affiliations:** 1 Graduate School Departments, Harbin Sport University, Heilongjiang, China; 2 College of Sport Science and Health, Harbin Sport University, Harbin, China

**Keywords:** cancer, cardiorespiratory fitness, exercise, fatigue, moderate-intensity physical activity, walking

## Abstract

**Objective:**

Walking is a simple and accessible form of aerobic exercise that may improve cardiorespiratory fitness (CRF) in cancer patients; however, its independent effects remain unclear. This systematic review and meta-analysis aimed to evaluate the effects of walking training on CRF, fatigue, dyspnea, safety, and adherence in adult cancer patients.

**Methods:**

A systematic search was conducted in 4 databases—Web of Science, Embase, PubMed, and Cochrane Library Database—covering the period from the inception of each database through November 28, 2025. The inclusion criteria were randomized controlled trials (RCTs) evaluating walking interventions for cancer patients. Priority was given to meta-analyses of outcome measures using a random-effects model. If the statistical heterogeneity is less than 40%, a fixed-effects model is used.

**Results:**

11 randomized controlled trials involving 649 participants were included. Compared with standard care or no intervention, walking training was associated with statistically significant improvements in the two outcomes: peak oxygen uptake (VO_2_ peak) (SMD = 0.56; 95% CI: [0.06, 1.06]; I² = 75%; τ²=0.28; P = 0.03) and fatigue (SMD = −0.45; 95% CI: [−0.71, −0.18]; I² = 32%; τ²=0; P = 0.001). In contrast, no statistically significant effects were observed for maximal oxygen uptake (VO_2_ max) (SMD = 0.20;95% CI: [−0.15, 0.54]; I² = 0%; P = 0.26), 6-Minute Walk Distance (6MWD) (MD = 53.97;95% CI: [−23.00, 130.93]; I² = 80%; τ²=2538;P = 0.17), and dyspnea (SMD = −0.18, 95% CI: [−0.47, 0.11]; I² = 0%; P = 0.23). No serious intervention-related adverse events were reported, and adherence rates ranged from 67% to 94%.

**Conclusion:**

Current evidence suggests that walking training may improve VO_2_ peak and reduce fatigue in cancer patients, while demonstrating acceptable short-term safety and adherence. However, these findings should be interpreted cautiously because the certainty of evidence remains low. Larger, high-quality randomized controlled trials with standardized intervention protocols and longer follow-up periods are needed to confirm these findings.

This study conducted a systematic literature search in accordance with the PRISMA (Preferred Reporting Items for Systematic Reviews and Meta-Analyses) guidelines. Prior to the search, the study protocol was prospectively registered on the international systematic review registry platform (PROSPERO, registration number: CRD420251140743) to ensure the scientific rigor and methodological soundness of this study.

**Systematic review registration:**

https://www.crd.york.ac.uk/prospero/, identifier CRD420251140743.

## 1 Introduction

Cancer is a major global health issue, with diagnosis rates rising year by year (1). In 2022, there were over 20 million new cancer cases worldwide, and the total number of cancer survivors has exceeded 50 million (2). It is projected that by 2050, approximately 35 million people will be newly diagnosed with cancer (2).

Impaired Cardiorespiratory fitness (CRF) is one of the most common complications in cancer patients undergoing antitumor therapy. For example, anthracycline-based chemotherapy and anti-HER2 targeted therapies can cause dose-dependent cardiotoxicity in cancer patients (3, 4), while radiation therapy can induce atherosclerosis (5), all of which lead to a decline in patients’ CRF. Peak oxygen uptake (VO_2_ peak), as an objective indicator of CRF, has been proven to be an independent predictor of all-cause mortality in cancer patients (6). Jones’s study indicates that for every 1 mL/(kg·min) increase in VO_2_ peak, the risk of all-cause mortality in lung cancer patients decreases by 4% (6).At the same time, a decline in CRF is often accompanied by cancer-related fatigue, dyspnea, and limitations in activities of daily living. These issues not only severely impair patients’ treatment adherence and long-term quality of life but may also increase the risk of discontinuing anticancer therapy and experiencing serious cardiovascular adverse events.

The benefits of exercise for cancer patients’ CRF have long been established (7). Exercise interventions can effectively improve physical function, reduce fatigue symptoms, alleviate anxiety and depression, and enhance overall quality of life (8).Authoritative organizations such as the American Society of Clinical Oncology, the American College of Sports Medicine, and the European Society for Medical Oncology have long issued guidelines recommending the incorporation of exercise training into the daily lives of all cancer patients and those in recovery (9–11). However, data indicates that as many as 70% of cancer patients still do not meet the recommended levels of physical activity, with participants citing various barriers to physical activity in their daily lives (12).

Walking is a simple, low-cost, and highly safe form of aerobic exercise that is widely applicable and easily accessible (13). Walking training is not restricted by location or equipment, and its intensity can be flexibly adjusted according to an individual’s physical condition (14), making it particularly suitable for patients with reduced physical fitness, the elderly, or cancer patients undergoing active treatment. For patients unable to access professionally supervised exercise programs, walking training serves as a vital option for home-based rehabilitation. A study by Martin et al. on physical activity choices among head and neck cancer patients revealed that 80% of patients chose walking as their exercise method (15).

However, existing studies exhibit significant heterogeneity in intervention protocols. Walking intensity ranged from low to high, intervention durations varied from several weeks to several months, frequency ranged from 3 to 7 times per week, and supervision methods included both supervised and self-directed home-based approaches (16–19).Patients with different cancer types, at different stages of treatment, and with varying baseline CRF levels may respond differently to walking training. Therefore, it is necessary to conduct a systematic evaluation of the effects of walking training on CRF in cancer patients using meta-analysis methods, and to explore potential moderating factors, thereby providing evidence-based support for the development of individualized exercise prescriptions.

In summary, this review follows the PRISMA guidelines to systematically retrieve relevant randomized controlled trials. It aims to explore the effects of walking training versus standard care or no intervention on cardiorespiratory fitness, fatigue, dyspnea, safety and adherence in adult cancer patients, so as to provide scientific evidence for formulating exercise prescriptions in cancer rehabilitation.

## 2 Research methods

### 2.1 Study registration

This study conducted a systematic literature search in accordance with the PRISMA (Preferred Reporting Items for Systematic Reviews and Meta-Analyses) guidelines. Prior to the search, the study protocol was prospectively registered on the international systematic review registry platform (PROSPERO, registration number: CRD420251140743) to ensure the scientific rigor and methodological soundness of this study.

### 2.2 Eligibility criteria

#### 2.2.1 Inclusion criteria

The inclusion criteria are as follows:

Randomized controlled trials published in full text. Study participants were adult cancer patients, with no restrictions on cancer stage, gender, or population type. The intervention consists of a walking exercise program. The control group must consist of standard care or a waiting list. The primary outcome measure is CRF and must include one of the following: VO_2_ peak, Maximal Oxygen Uptake (VO_2_ max), or 6-Minute Walk Distance (6MWD). Secondary outcome measures include heart rate, fatigue, dyspnea severity, adverse events, and adherence.

#### 2.2.2 Exclusion criteria

Exclusion criteria are as follows:

Non-adult cancer patients. Meta-analyses, reviews, guidelines, expert opinions, and articles that are not randomized controlled trials. Studies in which the intervention consists of a multimodal exercise program, or studies that include walking training but cannot isolate the independent effect of walking on outcome measures.

### 2.3 Information sources

This study carried out a comprehensive literature search in Web of Science, Embase, PubMed, and the Cochrane Library. The search was conducted on November 28, 2025, with no language restrictions. The search utilized Medical Subject Headings (MeSH) terms and free-text terms, employing the following search strategy: (TS= (Walking/Aerobic/home exercise-related terms) AND (TS= (cancer/tumor-related terms) AND (TS= (CRF-related terms)), and tailored search queries for each database(Web of Science: Topic; Embase: Emtree + title/abstract; Pubmed: MeSh + Title/Abstract; Cochrane Library: Mesh + Title/Abstract/Keyword). Manual searches were also conducted, and relevant review articles were manually traced to avoid omitting eligible studies, further ensuring the comprehensiveness and systematic nature of the literature search. The detailed search strategies and the exact search strings employed for each database are available in **Supplementary Material 1**.

### 2.4 Study selection

This study employed a standardized literature screening process to ensure study quality. First, all documents retrieved from the databases were imported into EndNote reference management software, and duplicate publications were identified and removed using the software’s duplication detection feature. After removing duplicate documents, 2 reviewers (Yu and Liu) independently and in parallel conducted an initial review of the titles and abstracts of the remaining documents, strictly following pre-established uniform criteria to identify candidate studies that might meet the inclusion criteria. For articles that passed the initial screening, full-text documents were obtained and subjected to a second, detailed evaluation, focusing on verifying key information such as study design, intervention measures, and outcome measures, thereby determining the final articles to be included in the analysis. The entire screening process was conducted independently by the 2 reviewers. To ensure the objectivity and reliability of the screening results, a third reviewer (Song) was consulted through discussion to resolve any disagreements that occurred at any stage.

### 2.5 Data extraction

To ensure the systematic and accurate extraction of data, we first created a standardized Excel spreadsheet to perform standardized data extraction for the included articles. The extracted data included: study characteristics (first author’s name, year of publication, country of study, study design, study duration); participant characteristics (number of participants in each group, gender distribution, age distribution, cancer type, treatment stage); intervention and control group characteristics (method, intensity, duration, frequency), and outcome measures (measurement and assessment tools, time points of testing, descriptive data of outcome measures). All extracted data were recorded and presented in the format of mean ± standard deviation (Mean ± SD). For data in the original literature that did not conform to this standard format (e.g., data presented as mean ± standard error, median and interquartile range, or reported only as a mean), we applied recognized statistical methods to standardize and convert the data to ensure consistency and comparability of data formats during the subsequent meta-analysis or descriptive summarization. For missing or unclear data, the corresponding authors were contacted whenever possible. When the necessary data could be retrieved or converted from the available information, they were extracted for quantitative analysis; otherwise, the study was excluded from the corresponding meta-analysis.

### 2.6 Risk of bias

This study used the Cochrane RoB 2.0 (Risk of Bias 2.0) tool to assess the risk of bias for all prespecified primary and secondary outcomes in each included randomized controlled trial. RoB 2.0 is the recommended tool for assessing the risk of bias in specific outcomes within systematic reviews. It features a clearer structure and a simpler, more efficient assessment process, enabling more accurate identification and quantification of potential sources of bias across all stages of study design, implementation, and reporting (20).The RoB 2.0 tool covers 5 key assessment domains: the randomization process, deviation from the assigned intervention, missing outcome data, outcome measurement, and selective reporting of results. Each domain includes a series of predefined questions, with response options including: Yes, No, Maybe, Not sure, and Unclear.

2 researchers independently assessed the risk of bias in the included studies using the Cochrane RoB 2.0 tool. Any disagreements during the assessment were resolved through discussion and consultation with a third researcher to ensure the objectivity and reliability of the results.

### 2.7 Statistical analysis

Meta-analysis was conducted using Review Manager (RevMan, version 5.4). For dichotomous outcome measures, the risk ratio (RR) and absolute risk difference (ARD) were used as the pooled effect sizes. For continuous outcome measures, the mean difference (MD) was used if studies employed homogeneous measurement methods, and the standardized mean difference (SMD) was used if heterogeneous measurement methods were employed. This study prioritized the random-effects model to calculate pooled effects and corresponding 95% confidence intervals, If the statistical heterogeneity is less than 40%, a fixed-effects model is used. defining a difference as statistically significant at a two-sided P < 0.05. Prior to data analysis, the scoring direction and measurement units of all outcome scales were standardized for consistency across studies. The results of the meta-analysis of outcome measures were presented as a forest plot, The criteria for determining heterogeneity were as follows: I² < 40% indicates low heterogeneity, 30%–60% indicates moderate heterogeneity,50%–90% indicates substantial heterogeneity, 75%–100% indicates considerable heterogeneity. Sensitivity analysis was performed using the leave-one-out method to verify the stability of the pooled results. If the number of included studies was ≥ 10, a meta-regression analysis was conducted to explore the sources of heterogeneity; and a funnel plot was used to assess the risk of publication bias in the included studies. Additionally, this study employs the Grading of Recommendations, Assessment, Development, and Evaluation (GRADE) approach to grade the quality of evidence for each outcome measure; a pilot search is conducted prior to formal analysis to confirm the required sample size of included studies, mitigate the risk of duplicate study registration, and preemptively manage study heterogeneity to prevent uncontrolled heterogeneity.

## 3 Results

### 3.1 Search results

**Figure 1:**
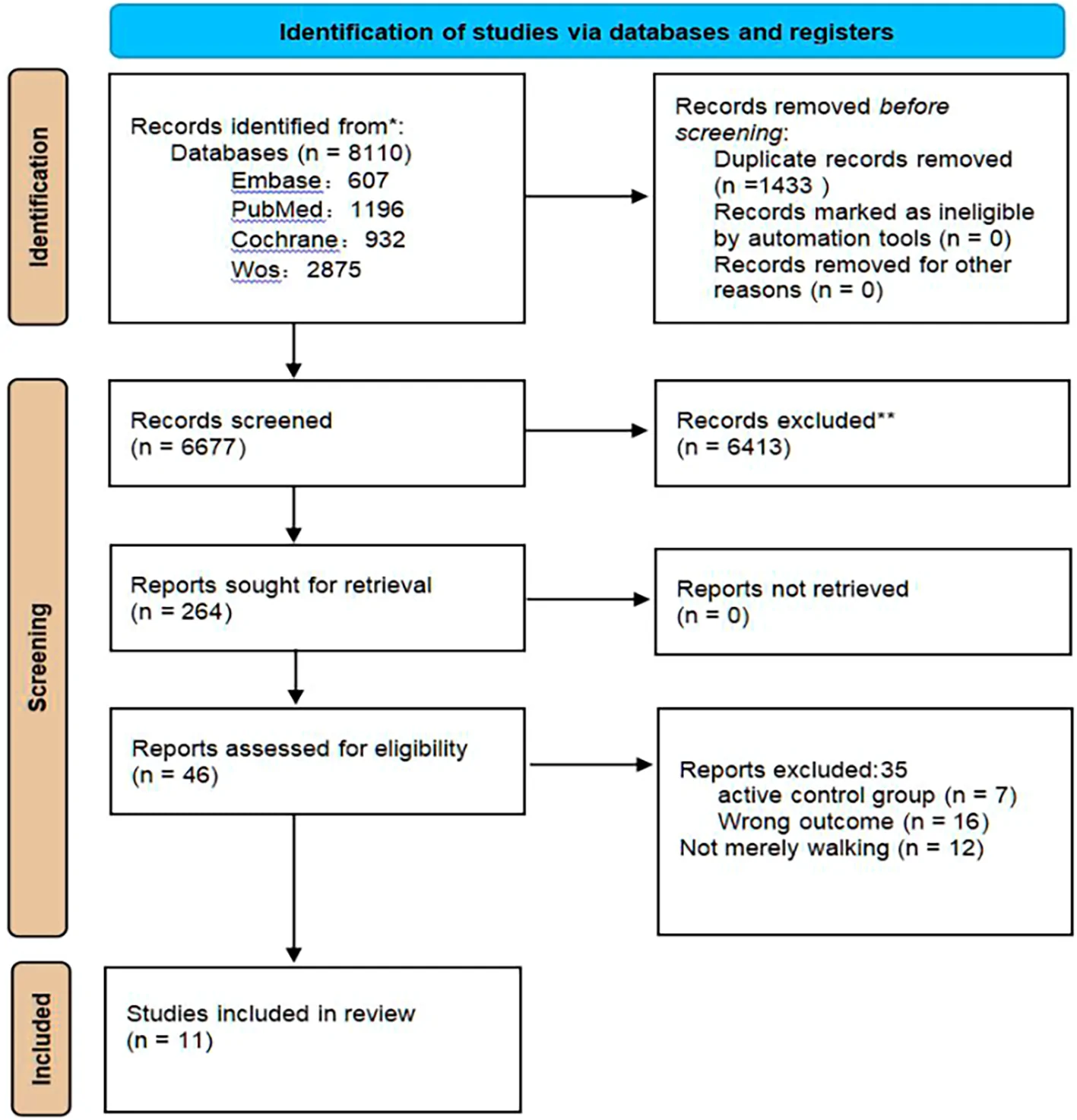
PRISMA flow diagram.

The screening process for this study is shown in **Figure 1**. The literature search was completed on November 28, 2025. A systematic retrieval was carried out in 4 major electronic databases. (Web of Science, Embase, PubMed, and Cochrane Library), yielding a total of 8,110 relevant records. After removing duplicates, 6,677 articles were included in the screening process. Following preliminary screening to exclude studies that clearly did not meet the inclusion criteria, 264 studies were identified as potentially eligible. These were then subjected to a detailed evaluation of their full-text content, and ultimately, 11 studies were confirmed to meet the inclusion criteria and were formally included in this meta-analysis for quantitative synthesis.

### 3.2 Study characteristics

The main characteristics of the studies that were included are presented in **Supplementary Material 1**.Intervention characteristics are summarized in **Supplementary Material 2**. This meta-analysis ultimately included 11 studies published between 2001 and 2024, conducted in the United States, Denmark, Iran, Canada, Ireland, Thailand, and Taiwan. The total sample size of the included studies was 649 cancer patients, with an average age range of 44.96 to 66.50 years. The included studies covered various cancer types, including 3 studies on breast cancer (16, 21, 22), 2 studies on prostate cancer (19, 23), 2 studies on colorectal cancer (17, 24), 1 study on lung cancer (25), and 1 study on esophageal cancer (26);There were also 2 studies on mixed cancers: one involving prostate, breast, and colorectal cancers (27); and another involving breast, prostate, lung, colorectal, and testicular cancers (18). Regarding the treatment phase, 7 studies were conducted during the participants’ treatment phase, including 5 during the chemotherapy or radiation therapy phase (21, 22, 24, 26, 27), 1 during hormone therapy (16), and 1 during active treatment (25). Of the remaining 4 studies, 2 were conducted in the post-surgical phase (17, 19), 1 after completion of active treatment (18), and 1 in the untreated phase (23). In addition, two included studies incorporated additional non-exercise intervention components alongside walking training. The study by Siripanya et al. (21) evaluated a home-based Buddhist walking meditation program, whereas Xu et al. (25) implemented a combined “walking plus dietary” intervention that included structured dietary guidance in addition to walking training.

### 3.3 Primary outcome measures

#### 3.3.1 VO_2_ peak and VO_2_ max

VO_2_ peak is defined as the highest oxygen uptake actually measured during an incremental exercise test (28). Among the included studies, 6 reported VO_2_ peak outcomes, including a total of 311 participants: 162 in the intervention group and 149 in the control group. 4 of these studies used incremental exercise testing combined with a respiratory gas analyzer to directly measure (19, 22–24), 1 study estimated it using the 1-mile Rockport Walking Test (RWT) (17), and 1 study used a mixed-method approach (27), where some participants underwent treadmill incremental exercise combined with respiratory gas analysis, while others were estimated using the 12-minute walk test.

**Figure 2:**
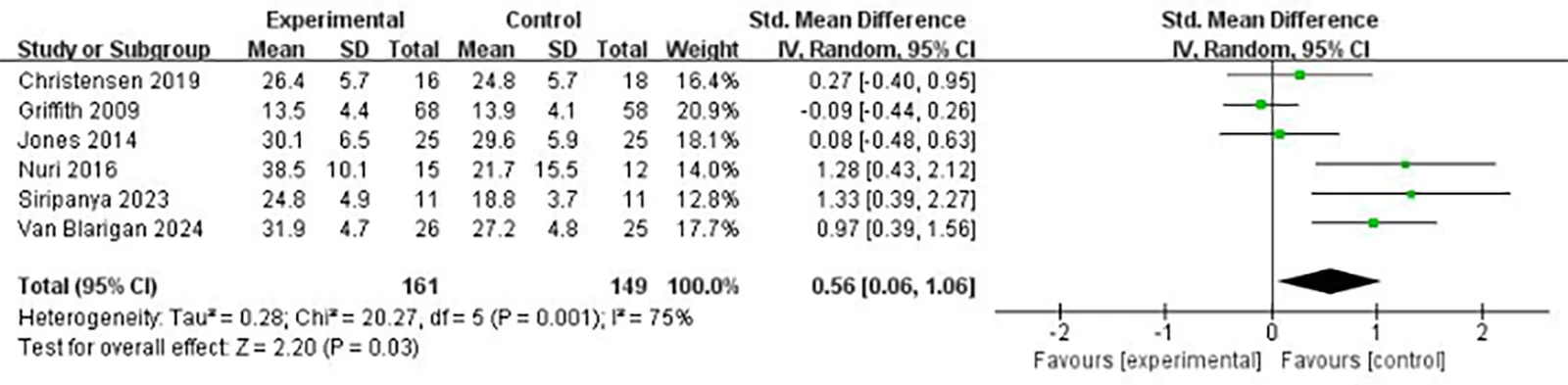
Forest Plot of VO2 Peak.

A meta-analysis using a random-effects model showed that walking training significantly improved VO_2_ peak in the intervention group (SMD = 0.56; 95% CI: [0.06, 1.06]; I² = 75%; τ²=0.28; P = 0.03), with high heterogeneity (I² = 75%; τ²=0.28) (**Figure 2**).

A leave-one-out sensitivity analysis indicated that no single study significantly altered the overall direction or magnitude of the pooled effect.

**Figure 3:**
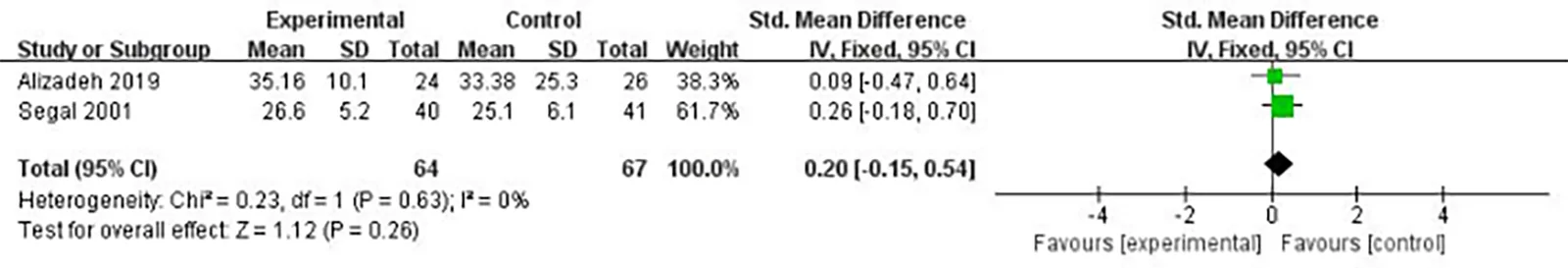
Forest Plot of VO_2_ Max.

For VO_2_ max, defined as the oxygen uptake at which a plateau is reached during incremental exercise (29), 2 studies involving the VO_2_ max measure (16, 21), with a total of 131 participants, showed no significant difference in the pooled effect size between the 2 groups following walking training intervention (SMD = 0.20;95% CI: [−0.15, 0.54]; I² = 0%; P = 0.26), with low heterogeneity between the 2 groups (**Figure 3**).

#### 3.3.2 6MWD

**Figure 4:**
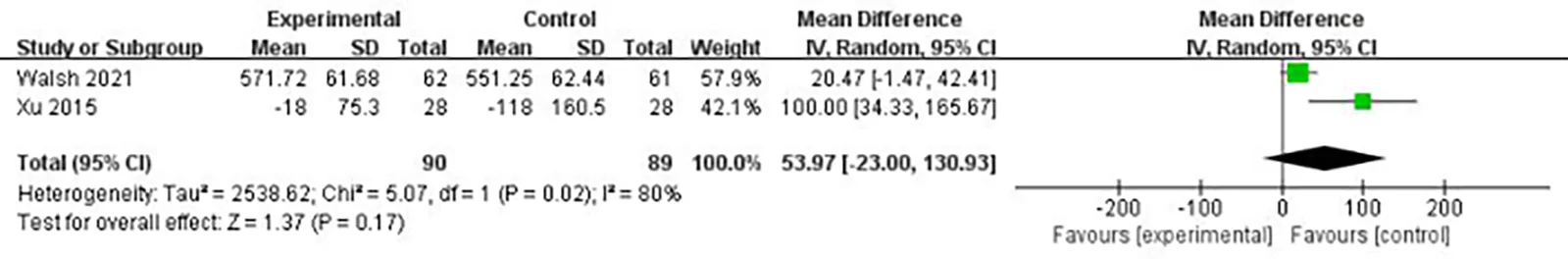
Forest Plot of 6MWT.

2 studies reported the 6MWD outcome measure (18, 26), involving a total of 167 participants. A meta-analysis using a random-effects model showed no significant difference between the intervention and control groups (MD = 53.97;95% CI: [−23.00, 130.93]; I² = 80%; τ²=2538; P = 0.17), and there was high heterogeneity (I² = 80%; τ²=2538) between the 2 studies (**Figure 4**).

### 3.4 Secondary outcomes

#### 3.4.1 Fatigue

**Figure 5:**
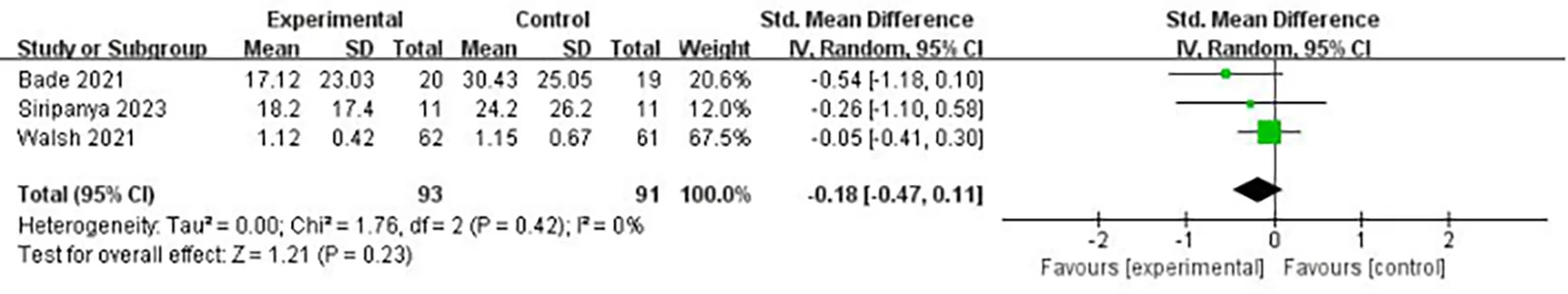
Forest Plot of Fatigue.

A total of 4 studies reported data on patient fatigue scores (18, 19, 22, 25), involving 228 participants: 116 in the intervention group and 112 in the control group. Higher scores indicate more severe fatigue. Due to low heterogeneity among studies, a meta-analysis using a fixed-effects model showed a significant difference between the 2 groups following intervention. Walking exercise significantly reduced patients’ fatigue scores and improved fatigue symptoms (SMD = −0.45; 95% CI: [−0.71, −0.18]; I² = 32%; τ²=0; P = 0.001), with low heterogeneity (**Figure 5**).

Sensitivity analysis indicated that the study by Jones et al. had a substantial impact on the pooled effect size (19).

#### 3.4.2 Dyspnea

**Figure 6:**
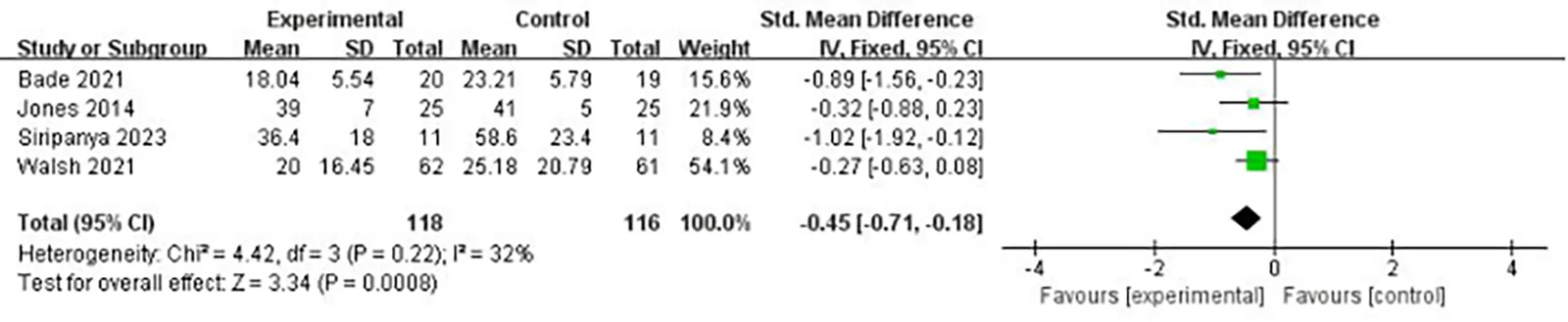
Forest Plot of Dyspnea.

3 studies reported on dyspnea outcomes (18, 22, 25), involving 178 participants. The intervention group comprised 91 participants, and the control group comprised 87 participants. Due to low heterogeneity among studies, a fixed-effects model was used for the meta-analysis. The results showed that, compared with the control group, the intervention did not significantly improve patients’ dyspnea symptoms; The difference between the two groups in dyspnea scale scores was not statistically significant (SMD = −0.18, 95% CI: [−0.47, 0.11]; I² = 0%; P = 0.23), indicating low heterogeneity (**Figure 6**).

Sensitivity analysis results indicated that the pooled effect size for dyspnea was highly sensitive to the study published by Siripanya et al. (22), which may be a key factor affecting the stability of the pooled effect size.

#### 3.4.3 Adverse events

Considerable heterogeneity was observed in adherence assessment approaches and reporting domains across studies; therefore, quantitative meta-analysis was not feasible, and data were synthesized narratively.

Among the studies included in this meta-analysis, a total of 5 reported the incidence of adverse events associated with walking interventions (19, 23–26). None of the studies reported serious adverse events. Van Blarigan et al. (23) reported 13 adverse events, including knee pain (2 cases), joint pain (5 cases), muscle pain (4 cases), and fatigue (2 cases); among these, 2 participants withdrew from the study due to adverse events, and 1 case involved intervention-related knee pain. In 3 studies by Bade, Christensen, and Xu (24–26), no intervention-related adverse events occurred during the walking exercise intervention.

#### 3.4.4 Adherence

A total of 8 studies reported on adherence.

Alizadeh et al. reported an 85% adherence rate among intervention group participants for each training session (16);

Bade et al. reported that, regarding device usage adherence, the intervention group provided usable step data for 90% of study weeks, with no instances of prolonged non-wear; regarding adherence to intervention goals, only 21% of study weeks met the targets; there were no cases of study withdrawal due to poor adherence (25);

Griffith et al. reported that adherence to the walking intervention in the intervention group was 67.6%, with participants walking an average of 117 minutes per week (27);

Jones et al. reported that overall intervention adherence was 79%, with 83% adherence to supervised training sessions and 72% adherence to home training sessions (19);

Siripanya et al. reported that the intervention adherence rate in the walking meditation group reached 92%; 11 out of 15 participants completed the entire intervention and follow-up, with a dropout rate of 26.7% (22);

Van Blarigan et al. reported that the intervention group completed 94% of the scheduled training sessions, with no participants withdrawing from the study due to poor adherence (23);

Walsh et al. reported that among the 54 participants in the intervention group who completed the goal-setting intervention, 69% (37/54) achieved at least 50% of their step goals (4 out of 8 target weeks) (18);

Xu et al. reported that participants in the walking program intervention group completed an average of 8.4 ± 3.6 walking sessions (12–15 scheduled sessions over 4–5 weeks), with an average adherence rate of 68% (26).

### 3.5 Risk of bias assessment

Detailed information on the risk of bias assessment can be found in **Supplementary Material 3**.

According to the Risk of Bias 2.0 (RoB 2.0) assessment, most studies were rated as having a low risk of bias in the domains of deviation from the intended intervention, missing outcome data, and measurement of outcomes. However, concerns were frequently identified in the domains of the randomization process and reporting of selection results. Due to the nature of walking interventions, none of the included trials were able to blind participants or intervention providers. Furthermore, among the 11 included studies, 4 employed assessor blinding (16, 19, 22, 24), while for the remaining 7 studies, the blinding status of outcome measurement could not be clearly determined due to insufficient reporting (17, 18, 21, 23, 25–27).Overall, most studies were rated as having some concerns regarding overall risk of bias, with only a few trials classified as having a low risk of bias.

### 3.6 Meta-regression and publication bias

The number of included studies per outcome ranged from 2 to 6 across all analyses. No outcome met the minimum threshold of 10 studies. Therefore, neither meta-regression nor publication bias assessment was performed in this review.

### 3.7 Assessment of evidence quality

We used the GRADE framework to assess the certainty of evidence across 5 domains included in the meta-analysis; details are provided in **Supplementary Material 4**.

The certainty of evidence for the 6 outcome measures ranged from low to very low, primarily due to substantial imprecision (wide confidence intervals) and inconsistency (moderate to high heterogeneity).

## 4 Discussion

### 4.1 Main findings

This meta-analysis examined the effects of walking training on CRF and related clinical outcomes in adult cancer patients. Previous meta-analyses in this field have primarily focused on multimodal aerobic exercise or combined resistance and aerobic exercise interventions (30, 31), with few studies examining walking training as a standalone intervention. To the authors’ knowledge, this is the first meta-analysis specifically evaluating the efficacy, safety, and adherence of walking training for CRF in cancer patients. This study strictly adhered to inclusion criteria, enrolling only randomized controlled trials, and imposed no restrictions on cancer type, treatment stage, or intervention delivery mode to comprehensively reflect the clinical outcomes of walking training. It is particularly important to emphasize that, limited by the number, sample size, and methodological quality of the included studies, all results of this meta-analysis should be interpreted as exploratory rather than confirmatory evidence, and its conclusions require further validation through subsequent large-scale, high-quality studies. Additionally, the VO_2_ peak outcome combined both directly measured and indirectly estimated assessment methods, which may have introduced confounding factors and resulted in considerable statistical heterogeneity, thereby further limiting our ability to isolate and confirm the independent therapeutic effect of walking training.

The results of this meta-analysis indicate that walking training can improve VO_2_ peak in cancer patients and effectively alleviate fatigue symptoms, but it did not produce statistically significant improvements in VO_2_ max, 6MWD, or dyspnea scores. Regarding safety, none of the included studies reported serious adverse events related to walking training, and overall intervention adherence ranged from 67% to 94%, However, due to limited data, conclusions on safety and adherence should be interpreted cautiously. In the VO_2_ peak analysis, a total of 6 studies involving 311 participants were included. Pooled results showed that walking training produced a moderate effect size (SMD = 0.56) for this measure, which reached statistical significance, suggesting that walking training can effectively improve VO_2_ peak in cancer patients. This finding is consistent with previous literature reporting that aerobic exercise can improve CRF in cancer patients (32, 33), providing exploratory directions for walking training as a means to promote CRF during cancer rehabilitation. It is worth noting that, although the pooled mean difference in 6MWD in this meta-analysis (MD: 53.97 m) has exceeded the minimum clinically important difference (MCID) for cancer patients (34), the heterogeneity for the 6MWD outcome was particularly severe. This extreme heterogeneity was mainly attributable to fundamental differences between the only two included studies in terms of population characteristics, intervention content, and outcome definitions. Therefore, this study cannot draw any reliable conclusions regarding the effect of walking training on 6MWD, and the pooled result can only suggest the presence of potential between-study differences.

### 4.2 Mechanism analysis

The mechanisms underlying the beneficial effects of walking training in cancer patients remain incompletely understood, because the included randomized controlled trials did not directly measure mechanistic biomarkers. Nevertheless, the observed improvements in VO_2_ peak and fatigue are biologically plausible and may be partly explained by the well-established adaptive responses to regular aerobic exercise. In skeletal muscle, moderate-intensity continuous training has been shown to increase mitochondrial volume density and citrate synthase activity, suggesting enhanced mitochondrial content and oxidative enzyme capacity (35). In addition, exercise can upregulate mitochondrial biogenesis-related signaling, including PGC-1α, thereby supporting oxidative phosphorylation and improving peripheral oxygen utilization (35, 36). These adaptations may help explain the improvement in cardiorespiratory fitness observed in the present meta-analysis.

Walking training may also contribute to the alleviation of cancer-related fatigue through anti-inflammatory and neurometabolic pathways. Previous studies have shown that exercise-induced PGC-1α signaling can increase kynurenine aminotransferase expression, reduce kynurenine accumulation in the brain, and potentially mitigate stress-related and fatigue-related symptoms (37). Exercise has also been associated with reduced pro-inflammatory cytokines and increased anti-inflammatory mediators, which may help interrupt inflammation-related fatigue pathways in cancer patients (38). Taken together, walking training may exert its effects through a combination of central and peripheral adaptations, including improved mitochondrial function, more efficient oxygen utilization, and attenuation of inflammation-related fatigue. However, because these mechanisms were not directly assessed in the included trials, this interpretation should be considered hypothesis-generating rather than confirmatory.

### 4.3 Clinical implications

Given its low cost and universal accessibility, structured walking training may be considered as a first-step exploratory exercise intervention for cancer patients’ rehabilitation, especially for elderly individuals or those in active treatment and early recovery phases who are unable to tolerate higher-intensity resistance or interval training. Despite the low certainty of evidence due to small sample sizes, high heterogeneity, and methodological limitations of included studies, our pooled results showed that walking training was associated with significant improvements in VO_2_ peak (the primary indicator of CRF) and significant reductions in cancer-related fatigue. These findings suggest that walking training may offer modest functional benefits for daily living activities in selected patient populations. When formulating individualized care plans, clinicians may consider prescribing tailored walking programs based on patients’ baseline functional capacity, comorbidities, and treatment status, with slow and gradual increases in training load. Consistent with the American Cancer Society guidelines recommending at least 150 minutes of moderate-intensity physical activity weekly for cancer patients (39). walking training provides a feasible and well-tolerated option to help patients meet these physical activity recommendations.

### 4.4 Limitations

When interpreting the results, one must give full consideration to the significant limitations that exist in this study.

The number of included studies was limited, and the spectrum of cancer types covered was narrow. In the meta-analysis of core CRF outcomes, only two studies were included for VO_2_ max and 6MWD, resulting in severely insufficient statistical power and making it difficult to accurately assess the true effects of walking training. Moreover, because each meta-analysis included fewer than 10 studies, meta-regression to explore sources of heterogeneity and formal assessment of publication bias were not feasible. This limits our ability to identify factors that may modify the effects of walking training and to rule out the potential influence of publication bias on our results (40).

The overall methodological quality of the included studies was low, with most studies rated as having some concern regarding overall risk of bias. Due to the nature of exercise interventions, none of the studies could achieve double-blinding of participants and intervention providers; some studies did not implement blinding for outcome assessors, which may have led to detection bias. Additionally, inadequate reporting of random sequence generation and allocation concealment in some studies further compromised the internal validity of the research.

The walking intervention protocols included in the study exhibited significant clinical heterogeneity. Core parameters—including intervention intensity, frequency, duration, supervision models, and progression protocols—lacked uniform standards, small sample sizes, making it difficult to determine optimal walking exercise prescription parameters. This is also a key factor contributing to the high heterogeneity among the studies. limits the reference value of the pooled effect estimates.

The included studies primarily focused on breast cancer patients, accounting for over 50% of the research; there was a severe lack of data on respiratory system tumors such as lung and esophageal cancer, as well as other rare cancer types. The narrow coverage of cancer subtypes limits the extrapolation of our findings to broader cancer populations.

The follow-up periods of the included studies were concentrated immediately after the intervention; only 1 study reported follow-up data at 3 months post-intervention. There is a lack of evidence regarding the long-term effects of walking training on CRF and quality of life in cancer patients, making it impossible to assess the sustainability of the intervention’s effects.

### 4.5 Implications for future research

Current evidence is limited by small sample sizes and inconsistent methodological quality. Subsequent studies should carry out large-scale, multi-center RCTs featuring prolonged follow-up durations to monitor how long intervention effects are sustained. Additionally, strict standardization of the randomization process and allocation concealment, along with blinding of outcome assessors, should be carried out to reduce potential bias and further improve methodological quality. Furthermore, future studies could incorporate biomarkers (such as inflammatory factors and mitochondrial function indicators) to explore the mechanisms of action of walking training, thereby providing more precise scientific evidence for optimizing intervention protocols.

## 5 Conclusion

This meta-analysis preliminarily investigated the effects of walking training on cardiorespiratory fitness and related clinical outcomes in cancer patients. The available results indicate that walking training may be associated with improvements in VO_2_ peak levels and alleviation of fatigue symptoms in cancer patients. No serious adverse events were reported during the intervention period, and the overall adherence rate fell within an acceptable range of 67% to 94%, demonstrating favorable short-term safety and patient acceptability. However, the analysis results for VO_2_ max, 6MWD, and dyspnea indicators show that the improvement effects of walking training have not yet reached statistical significance. It is important to note that the quality and certainty of evidence for all outcome measures are low, which may stem from methodological limitations, clinical heterogeneity, and imprecision of results. Therefore, no strong clinical recommendation can be formulated at this time. Future high-quality studies are needed to validate these findings and optimize exercise prescriptions for patients with different cancer types and treatment backgrounds.

## Data Availability

The original contributions presented in the study are included in the article/**Supplementary Material**. Further inquiries can be directed to the corresponding author.
