# Supplementary material for: A systematic review and meta-analysis of the effects of walking training on cardiorespiratory fitness in cancer patients
Source: Front Oncol. 2026 Jun 26;16:1852397. doi: 10.3389/fonc.2026.1852397 (PMC13349785; doi:10.3389/fonc.2026.1852397)
Supplement: Supplementary file 1 [file Table1.docx]

## **Supplementary S1：**Search terms and search strategies

## Pubmed

**#4**

Search: ((cancer[Title/Abstract] OR cancers[Title/Abstract] OR malignant neoplasia[Title/Abstract] OR malignant neoplastic disease[Title/Abstract] OR malignant tumor[Title/Abstract] OR malignant tumour[Title/Abstract] OR neoplasia, malignant[Title/Abstract] OR neoplasmic malignancy[Title/Abstract] OR neoplastic malignancy[Title/Abstract] OR oncologic malignancy[Title/Abstract] OR oncological malignancy[Title/Abstract] OR tumor, malignant[Title/Abstract] OR tumoral malignancy[Title/Abstract] OR tumorous malignancy[Title/Abstract] OR tumour, malignant[Title/Abstract] OR malignant neoplasm[Title/Abstract] OR carcinoma[Title/Abstract] OR carcinomas[Title/Abstract] OR malignancy[Title/Abstract] OR neoplastic disease[Title/Abstract] OR cancerous growth[Title/Abstract] OR solid tumor[Title/Abstract] OR solid neoplasm[Title/Abstract] OR hematologic malignancy[Title/Abstract] OR hematologic cancer[Title/Abstract] OR leukemia[Title/Abstract] OR lymphoma[Title/Abstract] OR sarcoma[Title/Abstract] OR myeloma[Title/Abstract] OR breast cancer[Title/Abstract] OR lung cancer[Title/Abstract] OR colorectal cancer[Title/Abstract] OR prostate cancer[Title/Abstract] OR liver cancer[Title/Abstract] OR ovarian cancer[Title/Abstract] OR esophageal cancer[Title/Abstract] OR pancreatic cancer[Title/Abstract] OR head neck cancer[Title/Abstract] OR skin cancer[Title/Abstract] OR brain tumor[Title/Abstract]) AND (walking[Title/Abstract] OR walking exercise[Title/Abstract] OR walking training[Title/Abstract] OR home-based exercise[Title/Abstract] OR ambulation[Title/Abstract] OR ambulatory exercise[Title/Abstract] OR physical activity[Title/Abstract] OR physical exercise[Title/Abstract] OR aerobic exercise[Title/Abstract] OR cardiovascular exercise[Title/Abstract] OR walking regimen[Title/Abstract] OR walking program[Title/Abstract] OR walking activity[Title/Abstract] OR walking intervention[Title/Abstract] OR treadmill walking[Title/Abstract] OR low-intensity walking[Title/Abstract] OR moderate-intensity walking[Title/Abstract] OR walking-based rehabilitation[Title/Abstract] OR walking for fitness[Title/Abstract] OR exercise walking[Title/Abstract] OR walking for health[Title/Abstract] OR walking therapy[Title/Abstract] OR exercise walking regimen[Title/Abstract] OR walking mobility[Title/Abstract] OR walking practice[Title/Abstract] OR walking challenge[Title/Abstract] OR outdoor walking[Title/Abstract] OR indoor walking[Title/Abstract] OR walking exercise[Title/Abstract] OR community walking[Title/Abstract] OR walking for wellness[Title/Abstract] OR active walking[Title/Abstract])) AND (cardiopulmonary fitness[Title/Abstract] OR cardiopulmonary function[Title/Abstract] OR cardiorespiratory fitness[Title/Abstract] OR cardiorespiratory function[Title/Abstract] OR cardiopulmonary performance[Title/Abstract] OR cardiorespiratory performance[Title/Abstract] OR cardiorespiratory capacity[Title/Abstract] OR aerobic capacity[Title/Abstract] OR aerobic fitness[Title/Abstract] OR exercise capacity[Title/Abstract] OR exercise tolerance[Title/Abstract] OR lung function[Title/Abstract] OR pulmonary function[Title/Abstract] OR respiratory function[Title/Abstract] OR ventilatory function[Title/Abstract] OR gas exchange capacity[Title/Abstract] OR maximal oxygen uptake[Title/Abstract] OR maximal oxygen consumption[Title/Abstract] OR VO2max[Title/Abstract] OR VO2max[Title/Abstract] OR peak oxygen consumption[Title/Abstract] OR peak VO2[Title/Abstract] OR peak oxygen uptake[Title/Abstract] OR ventilatory threshold[Title/Abstract] OR anaerobic threshold[Title/Abstract] OR oxygen uptake kinetics[Title/Abstract] OR oxygen diffusion capacity[Title/Abstract] OR diffusing capacity[Title/Abstract] OR ventilatory efficiency[Title/Abstract] OR minute ventilation[Title/Abstract] OR tidal volume[Title/Abstract] OR VO2peak[Title/Abstract] OR cardiorespiratory endurance[Title/Abstract] OR cardiac output[Title/Abstract] OR stroke volume[Title/Abstract] OR heart‐lung interaction[Title/Abstract] OR cardiopulmonary exercise test[Title/Abstract] OR CPET[Title/Abstract] OR cardiopulmonary exercise capacity[Title/Abstract])

*1,196*

**#3**

Search: cardiopulmonary fitness[Title/Abstract] OR cardiopulmonary function[Title/Abstract] OR cardiorespiratory fitness[Title/Abstract] OR cardiorespiratory function[Title/Abstract] OR cardiopulmonary performance[Title/Abstract] OR cardiorespiratory performance[Title/Abstract] OR cardiorespiratory capacity[Title/Abstract] OR aerobic capacity[Title/Abstract] OR aerobic fitness[Title/Abstract] OR exercise capacity[Title/Abstract] OR exercise tolerance[Title/Abstract] OR lung function[Title/Abstract] OR pulmonary function[Title/Abstract] OR respiratory function[Title/Abstract] OR ventilatory function[Title/Abstract] OR gas exchange capacity[Title/Abstract] OR maximal oxygen uptake[Title/Abstract] OR maximal oxygen consumption[Title/Abstract] OR VO2max[Title/Abstract] OR VO2max[Title/Abstract] OR peak oxygen consumption[Title/Abstract] OR peak VO2[Title/Abstract] OR peak oxygen uptake[Title/Abstract] OR ventilatory threshold[Title/Abstract] OR anaerobic threshold[Title/Abstract] OR oxygen uptake kinetics[Title/Abstract] OR oxygen diffusion capacity[Title/Abstract] OR diffusing capacity[Title/Abstract] OR ventilatory efficiency[Title/Abstract] OR minute ventilation[Title/Abstract] OR tidal volume[Title/Abstract] OR VO2peak[Title/Abstract] OR cardiorespiratory endurance[Title/Abstract] OR cardiac output[Title/Abstract] OR stroke volume[Title/Abstract] OR heart‐lung interaction[Title/Abstract] OR cardiopulmonary exercise test[Title/Abstract] OR CPET[Title/Abstract] OR cardiopulmonary exercise capacity[Title/Abstract]

*244,395*

**#2**

Search: walking[Title/Abstract] OR walking exercise[Title/Abstract] OR walking training[Title/Abstract] OR home-based exercise[Title/Abstract] OR ambulation[Title/Abstract] OR ambulatory exercise[Title/Abstract] OR physical activity[Title/Abstract] OR physical exercise[Title/Abstract] OR aerobic exercise[Title/Abstract] OR cardiovascular exercise[Title/Abstract] OR walking regimen[Title/Abstract] OR walking program[Title/Abstract] OR walking activity[Title/Abstract] OR walking intervention[Title/Abstract] OR treadmill walking[Title/Abstract] OR low-intensity walking[Title/Abstract] OR moderate-intensity walking[Title/Abstract] OR walking-based rehabilitation[Title/Abstract] OR walking for fitness[Title/Abstract] OR exercise walking[Title/Abstract] OR walking for health[Title/Abstract] OR walking therapy[Title/Abstract] OR exercise walking regimen[Title/Abstract] OR walking mobility[Title/Abstract] OR walking practice[Title/Abstract] OR walking challenge[Title/Abstract] OR outdoor walking[Title/Abstract] OR indoor walking[Title/Abstract] OR walking exercise[Title/Abstract] OR community walking[Title/Abstract] OR walking for wellness[Title/Abstract] OR active walking[Title/Abstract]

*317,990*

**#1**

Search: cancer[Title/Abstract] OR cancers[Title/Abstract] OR malignant neoplasia[Title/Abstract] OR malignant neoplastic disease[Title/Abstract] OR malignant tumor[Title/Abstract] OR malignant tumour[Title/Abstract] OR neoplasia, malignant[Title/Abstract] OR neoplasmic malignancy[Title/Abstract] OR neoplastic malignancy[Title/Abstract] OR oncologic malignancy[Title/Abstract] OR oncological malignancy[Title/Abstract] OR tumor, malignant[Title/Abstract] OR tumoral malignancy[Title/Abstract] OR tumorous malignancy[Title/Abstract] OR tumour, malignant[Title/Abstract] OR malignant neoplasm[Title/Abstract] OR carcinoma[Title/Abstract] OR carcinomas[Title/Abstract] OR malignancy[Title/Abstract] OR neoplastic disease[Title/Abstract] OR cancerous growth[Title/Abstract] OR solid tumor[Title/Abstract] OR solid neoplasm[Title/Abstract] OR hematologic malignancy[Title/Abstract] OR hematologic cancer[Title/Abstract] OR leukemia[Title/Abstract] OR lymphoma[Title/Abstract] OR sarcoma[Title/Abstract] OR myeloma[Title/Abstract] OR breast cancer[Title/Abstract] OR lung cancer[Title/Abstract] OR colorectal cancer[Title/Abstract] OR prostate cancer[Title/Abstract] OR liver cancer[Title/Abstract] OR ovarian cancer[Title/Abstract] OR esophageal cancer[Title/Abstract] OR pancreatic cancer[Title/Abstract] OR head neck cancer[Title/Abstract] OR skin cancer[Title/Abstract] OR brain tumor[Title/Abstract]

## WOS

**#4**

#3 AND #2 AND #1

*5,381*

**#3**

cardiopulmonary fitness OR cardiopulmonary function OR cardiorespiratory fitness OR cardiorespiratory function OR cardiopulmonary performance OR cardiorespiratory performance OR cardiorespiratory capacity OR aerobic capacity OR aerobic fitness OR exercise capacity OR exercise tolerance OR lung function OR pulmonary function OR respiratory function OR ventilatory function OR gas exchange capacity OR maximal oxygen uptake OR maximal oxygen consumption OR VO2max OR VO₂max OR peak oxygen consumption OR peak VO₂ OR peak oxygen uptake OR ventilatory threshold OR anaerobic threshold OR oxygen uptake kinetics OR oxygen diffusion capacity OR diffusing capacity OR ventilatory efficiency OR minute ventilation OR tidal volume OR VO₂peak OR cardiorespiratory endurance OR cardiac output OR stroke volume OR heart‑lung interaction OR cardiopulmonary exercise test OR CPET OR cardiopulmonary exercise capacity (Topic)

*604,724*

**#2**

walking OR walking exercise OR walking training OR home-based exercise OR ambulation OR ambulatory exercise OR physical activity OR physical exercise OR aerobic exercise OR cardiovascular exercise OR walking regimen OR walking program OR walking activity OR walking intervention OR treadmill walking OR low-intensity walking OR moderate-intensity walking OR walking-based rehabilitation OR walking for fitness OR exercise walking OR walking for health OR walking therapy OR exercise walking regimen OR walking mobility OR walking practice OR walking challenge OR outdoor walking OR indoor walking OR walking exercise OR community walking OR walking for wellness OR active walking (Topic)

*914,207*

**#1**1

cancer OR cancers OR malignant neoplasia OR malignant neoplastic disease OR malignant tumor OR malignant tumour OR neoplasia, malignant OR neoplasmic malignancy OR neoplastic malignancy OR oncologic malignancy OR oncological malignancy OR tumor, malignant OR tumoral malignancy OR tumorous malignancy OR tumour, malignant OR malignant neoplasm OR carcinoma OR carcinomas OR malignancy OR neoplastic disease OR cancerous growth OR solid tumor OR solid neoplasm OR hematologic malignancy OR hematologic cancer OR leukemia OR lymphoma OR sarcoma OR myeloma OR breast cancer OR lung cancer OR colorectal cancer OR prostate cancer OR liver cancer OR ovarian cancer OR esophageal cancer OR pancreatic cancer OR head neck cancer OR skin cancer OR brain tumor (Topic)

*5,149,274*

## Cochrane

**#4**

#3 AND #2 AND #1

*932*

**#3**

cardiopulmonary fitness（MeSH descriptor）OR cardiopulmonary function OR cardiorespiratory fitness OR cardiorespiratory function OR cardiopulmonary performance OR cardiorespiratory performance OR cardiorespiratory capacity OR aerobic capacity OR aerobic fitness OR exercise capacity OR exercise tolerance OR lung function OR pulmonary function OR respiratory function OR ventilatory function OR gas exchange capacity OR maximal oxygen uptake OR maximal oxygen consumption OR VO2max OR VO₂max OR peak oxygen consumption OR peak VO₂ OR peak oxygen uptake OR ventilatory threshold OR anaerobic threshold OR oxygen uptake kinetics OR oxygen diffusion capacity OR diffusing capacity OR ventilatory efficiency OR minute ventilation OR tidal volume OR VO₂peak OR cardiorespiratory endurance OR cardiac output OR stroke volume OR heart‑lung interaction OR cardiopulmonary exercise test OR CPET OR cardiopulmonary exercise capacity（ti，ab，kw）

*106818*

**#2**

walking（MeSH descriptor） OR walking exercise OR walking training OR home-based exercise OR ambulation OR ambulatory exercise OR physical activity OR physical exercise OR aerobic exercise OR cardiovascular exercise OR walking regimen OR walking program OR walking activity OR walking intervention OR treadmill walking OR low-intensity walking OR moderate-intensity walking OR walking-based rehabilitation OR walking for fitness OR exercise walking OR walking for health OR walking therapy OR exercise walking regimen OR walking mobility OR walking practice OR walking challenge OR outdoor walking OR indoor walking OR walking exercise OR community walking OR walking for wellness OR active walking（ti，ab，kw）

*140011*

**#1**

cancer （MeSH descriptor）OR tumour（MeSH descriptor） OR cancers OR malignant neoplasia OR malignant neoplastic disease OR malignant tumor OR malignant tumour OR neoplasia, malignant OR neoplasmic malignancy OR neoplastic malignancy OR oncologic malignancy OR oncological malignancy OR tumor, malignant OR tumoral malignancy OR tumorous malignancy OR tumour, malignant OR malignant neoplasm OR carcinoma OR carcinomas OR malignancy OR neoplastic disease OR cancerous growth OR solid tumor OR solid neoplasm OR hematologic malignancy OR hematologic cancer OR leukemia OR lymphoma OR sarcoma OR myeloma OR breast cancer OR lung cancer OR colorectal cancer OR prostate cancer OR liver cancer OR ovarian cancer OR esophageal cancer OR pancreatic cancer OR head neck cancer OR skin cancer OR brain tumor （ti，ab，kw）

*275876*

## Embase

**#4**

#3 AND #2 AND #1

*2121*

**#3**

cardiopulmonary fitness（/exp）OR cardiopulmonary function OR cardiorespiratory fitness OR cardiorespiratory function OR cardiopulmonary performance OR cardiorespiratory performance OR cardiorespiratory capacity OR aerobic capacity OR aerobic fitness OR exercise capacity OR exercise tolerance OR lung function OR pulmonary function OR respiratory function OR ventilatory function OR gas exchange capacity OR maximal oxygen uptake OR maximal oxygen consumption OR VO2max OR VO₂max OR peak oxygen consumption OR peak VO₂ OR peak oxygen uptake OR ventilatory threshold OR anaerobic threshold OR oxygen uptake kinetics OR oxygen diffusion capacity OR diffusing capacity OR ventilatory efficiency OR minute ventilation OR tidal volume OR VO₂peak OR cardiorespiratory endurance OR cardiac output OR stroke volume OR heart‑lung interaction OR cardiopulmonary exercise test OR CPET OR cardiopulmonary exercise capacity（ti，ab，kw）

*368878*

**#2**

walking（/exp）OR walking exercise OR walking training OR home-based exercise OR ambulation OR ambulatory exercise OR physical activity OR physical exercise OR aerobic exercise OR cardiovascular exercise OR walking regimen OR walking program OR walking activity OR walking intervention OR treadmill walking OR low-intensity walking OR moderate-intensity walking OR walking-based rehabilitation OR walking for fitness OR exercise walking OR walking for health OR walking therapy OR exercise walking regimen OR walking mobility OR walking practice OR walking challenge OR outdoor walking OR indoor walking OR walking exercise OR community walking OR walking for wellness OR active walking（ti，ab，kw）

*447065*

**#1**

cancer（/exp）OR tumour（/exp） OR cancers OR malignant neoplasia OR malignant neoplastic disease OR malignant tumor OR malignant tumour OR neoplasia, malignant OR neoplasmic malignancy OR neoplastic malignancy OR oncologic malignancy OR oncological malignancy OR tumor, malignant OR tumoral malignancy OR tumorous malignancy OR tumour, malignant OR malignant neoplasm OR carcinoma OR carcinomas OR malignancy OR neoplastic disease OR cancerous growth OR solid tumor OR solid neoplasm OR hematologic malignancy OR hematologic cancer OR leukemia OR lymphoma OR sarcoma OR myeloma OR breast cancer OR lung cancer OR colorectal cancer OR prostate cancer OR liver cancer OR ovarian cancer OR esophageal cancer OR pancreatic cancer OR head neck cancer OR skin cancer OR brain tumor（ti，ab，kw）

*2584302*

**Supplementary S2：**Characteristics of the included studies

| **Autor Year Country** | **Study Design** | **Participants Sample Size Male/Female Age *Cancer Type Phase of Therapy** | **Intervention** | **Control** | **Outcomes and Measurement Tools** | **Endpoints** |
| --- | --- | --- | --- | --- | --- | --- |
| Alizadeh  2019  Iran | 1. arm   RCT  single-center | 50 (IG 24/CG 26)  All female  IG (49.2 ± 9.7)/CG (48.42 ± 7.54)  Breast cancer  Undergoing hormone therapy | Interval Walking Training | Usual care | VO2max(1-mile Rockport Walk Test estimation) | Post-12-week intervention |
| Bade  2021  US | 2-arm  RCT,  single-center | 40 (IG 20/CG 20)  Female (IG 8/CG 18)  Male (IG 12/CG 2)  IG (66.5 ±7.3)/CG (63.2 ±9.8)  Lung cancer  During treatment：34  Post-treatment：6 | Walking Training | Usual care | Degree of dyspnea (MMRC) | Post-12-week intervention |
| Christensen  2019  Denmark | 2-arm  RCT  single-center | 34 (IG 16/CG 18)  Female (IG 9/CG 12)  Male (IG 11/CG 7)  IG (57.8 ±10.4)/CG (60.3±8.9)  Colorectal cancer  Post-surgery | Interval Walking Training | Usual care | VO2peak(Incremental Exercise Test with gas analysis)  heart rate | Post-12-week intervention |
| Griffith  2009  US | 2-arm  RCT  single-center | 126 (IG 68/CG 58)  Female (IG 41/CG 36)  Male (IG 27/CG 22)  IG (59.8 ±10.8)/CG (60.6±10.8)  Prostate Cancer、Breast cancer、Colorectal cancer、Other  During treatment | Walking Training | Usual care | VO2peak(Treadmill with gas analysis) | Post-treatment |

**Supplementary S2：***cont*

| **Autor Year Country** | **Study Design** | **Participants Sample Size Male/Female Age *Cancer Type Phase of Therapy** | **Intervention** | **Control** | **Outcomes and Measurement Tools** | **Endpoints** |
| --- | --- | --- | --- | --- | --- | --- |
| Jones  2014  US | 2-arm  RCT  single-center | 50 (IG 25/CG 25)  All male  IG (61 ±5)/CG (58±8)  Prostate Cancer  Post-surgery | Walking Training | Usual care | VO2peak(Treadmill with gas analysis/ 12MWT estimation) heart rate | Post-6-month intervention |
| Nuri  2016  Iran | Arm  RCT  single-center | 27 (IG 15/CG 12)  All male  IG (51.6 ± 11.3)/CG (51.6 ± 11.3)  Colorectal cancer  Post-surgery | Walking Training | Usual care | VO2peak(1-mile Rockport Walk Test estimation) | Post-8-week intervention/9 weeks |
| Segal  2001  Canada | Arm  RCT  single-center | 81 (IG1 40/IG2 12/CG 41)  All female  IG (51.0 ±8.7)/IG2(51.4±8.7)/CG (50.3 ± 8.7)  Breast cancer  During treatment | Walking Training | Usual care | VO2max(mCAFT) | Post-26-week intervention |
| Siripanya  2023  US | 1. Arm   RCT  single-center | 22 (IG 11/CG 11)  All female  IG (45 ±8)/CG (44.9 ± 8)  Breast cancer  During treatment | Walking Training and meditation | Usual care | VO2peak(Treadmill with gas analysis)  heart rate  Cardiac Output(transthoracic electrical bioimpedance meter) | Post-12-week intervention |
| Van Blarigan  2024  US | 2-arm  RCT  single-center | 61 (IG 26/CG 25)  All male  IG (61.2 ±5.8)/CG (64.7±7.4)  Prostate Cancer  No treatment | Walking Training | Usual care | VO2peak(CPET) | Post-16-week intervention |

**Supplementary S2：** *cont*

| **Autor Year Country** | **Study Design** | **Participants Sample Size Male/Female Age *Cancer Type Phase of Therapy** | **Intervention** | **Control** | **Outcomes and Measurement Tools** | **Endpoints** |
| --- | --- | --- | --- | --- | --- | --- |
| Walsh  2021  Ireland | 2-arm  RCT  single-center | 101 (IG 53/CG 48)  Female (IG 42/CG 49)  Male (IG 12/CG 4)  IG (55.6 ±8.0)/CG (59.2 ±7.6)  Breast cancer、Prostate Cancer、Lung cancer、Colorectal cancer、Testicular Cancer  Post-treatment | Walking Training | Usual care | 6MWT  heart rate  Degree of dyspnea | Post-12-week intervention/24 weeks |
| Xu  2015  Taiwan | 2-arm  RCT  single-center | 56 (IG 28/CG 28)  Female (IG 2/CG 2)  Male (IG 26/CG 26)  IG (58.1±9.1)/CG (61.1±9.0)  Esophageal Cancer  During treatment | Walking Training and Dietary advice | Usual care and Dietary advice | 6MWT | Post-treatment |

Table 1. Characteristics of the included studies

* Age is presented as mean ± SD.

**Supplementary S3：** Intervention Description

| **Study** | **Supervision Description** | | **Frequency** | **Intervention Description** | **Length of**  **Intervention** |
| --- | --- | --- | --- | --- | --- |
| Alizadeh/2019 | All training sessions were supervised(36) | | three times per week | Each 38-min session: 5-min warm-up, 5-min rest, 16-min HIIT (4×4-min uphill walking at 90–95% HRmax), and 12-min active recovery (4×3-min uphill walking at 50–70% HRmax) | 12 weeks |
| Bade/2021 | Continuous remote monitoring of daily step counts, with automated SMS reminders and goal guidance | | every day | Increase by 400 steps/day weekly from baseline (first-week average) until reaching 10,000 steps/day; maintain previous target if unmet | 12 weeks |
| Christensen/2019 | unsupervised | | 150 minutes per week | Repeat the cycle of 3 minutes of slow walking followed by 3 minutes of fast walking | 12 weeks |
| Griffith/2009 | unsupervised | | five times per week | Brisk walking for 20 to 30 minutes, followed by 5 minutes of slow walking | 5-35 weeks |
| Jones/2014 | At least 3 training sessions per week were supervised, and participants could make their own choice for the remaining 2 sessions(≥78) | | five times per week | Supervised walking sessions of 30–45 minutes each at 55%–100% VO₂peak | 6 months |
| Nuri/2016 | unsupervised | | three times per week | 45-minute walking sessions at 50%–60% heart rate | 8 weeks |
| Segal/2001 | | No supervision was provided for the self-directed exercise group, with the supervised exercise group completing 78 supervised training sessions in total | five times per week | A progressive walking program performed at 50%–60%VO₂max | 26 weeks |
| Siripanya/2023 | | unsupervised | three times per week | Weeks 1–6: 30 min walking at 41–50% HRR (3×10-min bouts, 3-min rest between).  Weeks 7–12: 45 min walking at 51–60% HRR (3×15-min bouts, 3-min rest between) | 12 weeks |
| Van Blarigan/2024 | | Continuous remote data monitoring | Begin with training three days per week. From week 5 onwards, increase to exercising four times per week | 20–60 minute walking sessions with progressive duration escalation, delivered at an intensity of 45%–80% VO₂peak | 16 weeks |
| Walsh/2021 | | Continuous remote data monitoring | every day | Remote step intervention: weekly SMS feedback with personalized 10% weekly step goal increase | 8 weeks |
| Xu/2015 | Nurse-supervised on-site walking training and weekly nutritional advice | | three times per week | Walking program: 5-min warm-up + 20-min corridor walking at an intensity of 60% HRmax | 4-5weeks |

**Supplementary S4：**Risk of Bias Assessment

**
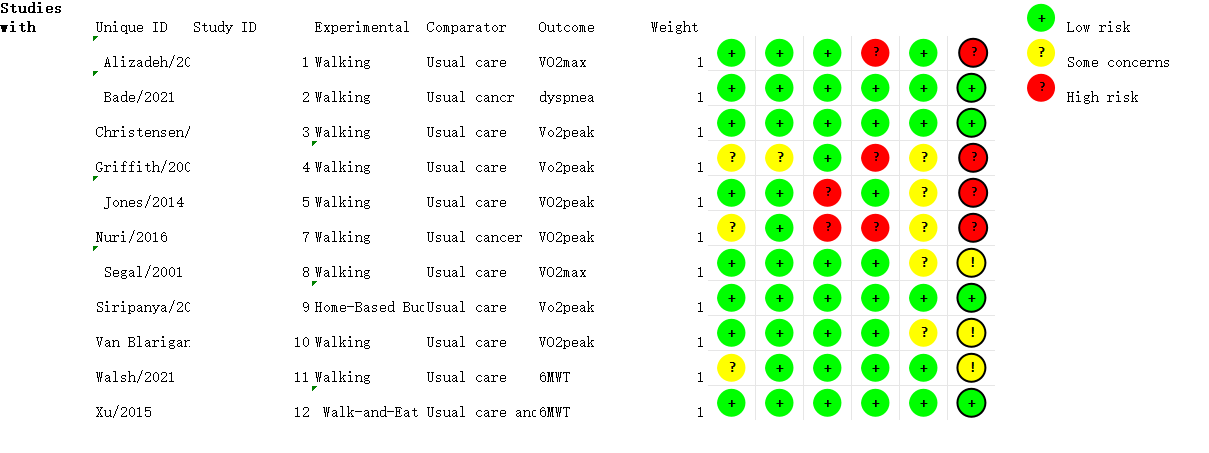
**

**Supplementary S5：**Quality of Evidence Assessment

**Author(s):**

**Question:** [Walking] compared to [Usual care] for [Vo2peak]

**Setting:**

**Bibliography:**

| **Certainty assessment** | | | | | | | **№ of patients** | | **Effect** | | **Certainty** | **Importance** |
| --- | --- | --- | --- | --- | --- | --- | --- | --- | --- | --- | --- | --- |
| **№ of studies** | **Study design** | **Risk of bias** | **Inconsistency** | **Indirectness** | **Imprecision** | **Other considerations** | **[Walking]** | **[Usual care]** | **Relative (95% CI)** | **Absolute (95% CI)** |  |  |
| **New outcome** | | | | | | | | | | | | |
|  |  |  |  |  |  |  |  |  | not estimable |  | - |  |
| **Vo2peak** | | | | | | | | | | | | |
| 6 | randomised trials | serious^a^ | serious^b^ | not serious | serious^c^ | none | 173 | 161 | - | SMD **0.25 higher** (0.03 higher to 0.47 higher) | ⨁◯◯◯ Very low^a,b,c^ | CRITICAL |
| **Vo2max** | | | | | | | | | | | | |
| 2 | randomised trials | not serious | not serious | not serious | serious^c^ | none | 64 | 67 | - | SMD **0.2 higher** (0.15 lower to 0.54 higher) | ⨁⨁⨁◯ Moderate^c^ | CRITICAL |
| **6MWD** | | | | | | | | | | | | |
| 2 | randomised trials | serious^a^ | serious^b^ | not serious | serious^c^ | none | 90 | 89 | - | MD **53.97 higher** (23 lower to 130.93 higher) | ⨁◯◯◯ Very low^a,b,c^ | CRITICAL |
| **dyspnea** | | | | | | | | | | | | |
| 3 | randomised trials | serious^a^ | not serious | not serious | serious^d^ | none | 103 | 99 | - | SMD **0.14 lower** (0.41 lower to 0.14 higher) | ⨁⨁◯◯ Low^a,d^ | CRITICAL |
| **fatigue** | | | | | | | | | | | | |
| 4 | randomised trials | serious^a^ | not serious | not serious | serious^d^ | none | 128 | 124 | - | SMD **0.39 lower** (0.64 lower to 0.14 lower) | ⨁⨁◯◯ Low^a,d^ | CRITICAL |

**CI:** confidence interval; **MD:** mean difference; **SMD:** standardised mean difference

#### Explanations

a. ROB: High risk

b. Substantial heterogeneity was observed between studies (I² ＞50).

c. The estimate had a wide confidence interval, indicating uncertainty in the effect size

d. The estimate of the effect size lacked precision

**Supplementary S6：**Sensitivity analysis

| **Excluded_study** | **Std. MD** | **Lower** | **Upper** | **CrI_Width** |
| --- | --- | --- | --- | --- |
| **VO_2_ peak** |  |  |  |  |
| **Christensen/2019** | **0.43** | **-0.16** | **1.02** | **1.18** |
| **Griffith/2009** | **0.51** | **-0.07** | **1.10** | **1.17** |
| **Jones/2014** | **0.47** | **-0.14** | **1.08** | **1.22** |
| **Nuri/2016** | **0.27** | **-0.23** | **0.78** | **1.01** |
| **Siripanya/2023** | **0.28** | **-0.22** | **0.78** | **1.00** |
| **Van Blarigan/2024** | **0.29** | **-0.23** | **0.82** | **1.05** |
| **Dyspnea** |  |  |  |  |
| **Bade/2021** | **-0.04** | **-0.35** | **0.27** | **0.62** |
| **Siripanya/2023** | **-0.13** | **-0.51** | **0.24** | **0.75** |
| **Walsh/2021** | **-0.26** | **-0.73** | **0.21** | **0.94** |
| **Fatigue** |  |  |  |  |
| **Bade/2021** | **-0.31** | **-0.58** | **-0.03** | **-0.91** |
| **Jones/2014** | **-0.41** | **-0.69** | **-0.13** | **-0.72** |
| **Siripanya/2023** | **-0.34** | **-0.60** | **-0.08** | **-0.68** |
| **Walsh/2021** | **-0.51** | **-0.86** | **-0.15** | **-1.01** |
